# New York Letter

**Published:** 1893-03

**Authors:** R. Harcourt Anderson

**Affiliations:** 260 West 21st Street, New York City


### *CORRESPONDENCE.*

---

New York, February 15, 1893.

TUBERCULOSIS.

As this is an age of hypodermatic medication, it is interesting to note the very varied experience of the practitioner. The Gibbes-Shurly method of treating tuberculosis modified by J. Blake White has been employed by a number of observers with successful results. It is doubtful however, if it is applicable to the greater number of the affected ; the dispensary patient is referred to particularly. For those not familiar with the method, mention of it will be made. The solution used is of iodide of gold and manganese in glycerine, each minim representing one-fiftieth grain of the salt, and from one half to three minims is the dose. A syringe is used which prevents contact of the piston with the solution. Before giving the injection a small pledget of cotton saturated with a mixture of chloriform and ether is applied to the skin, producing local anæsthesia and preventing irritation. Dr. E. Styles Potter, one of the attending physicians to the DeMilt Dispensary, has recently employed this method with results which are not flattering. The belief, however, is expressed that under certain conditions, as where proper food and hygiene is possible, it would show much better results.

The disadvantages of the treatment in dispensary practice are about as follows : 1. Poor food and bad hygiene of the patient. 2. Reluctance of weak patients to undergo the hypodermatic operation. 3. The injections should be made at night, and once every forty-eight hours ; this is impossible when the patient can only be seen every second day. 4. Diminution in the weight of the patient for the first two or three weeks, but this is counteracted later on by increase in weight. 5. In susceptible cases there occurs itching of skin, profuse diarrhœa, coryza and marked anorexia. When this occurs it is advisable to stop the treatment and depend on other means of helping the patient. 6. In selected cases it undoubtedly injures the general health. Some of the advantages to be derived are as follows : 1. Marked improvement in general health, *i. e.*, the patient ate heartily, slept well, night sweats ceased, the pyrexia diminished and a remarkable improvement in the catarrhal condition was brought about 2. The treatment can be carried out under the eye.

The mistake of commencing with large doses should not be made ; one half minim is the proper dose to commence with, and increase gradually, watching the effect closely and aiding the patient by every other means possible. Clinically considered, the drug does not seem to have any selective action on the tubercular processes proper, the effect being produced through the organism as a whole.

In a recent article Solis-Cohen with fine insight and remarkable precision urges that the proper use be made of air, water and sunshine ; the fact that exposure to sunlight for three days will kill the bacillus tuberculosis should always be kept in mind. Water properly used increases the expansive power of the smallest chest ; food, air, sunshine and water should be placed, and rightly placed, first as therapeutic agents. Intellectual and mental influences should not be forgotten, but how many physicians give patients exact words in regard to these. One of the dangers which lurk in our metropolis is the wretched supervision of food by inspectors. This particularly refers to raw meats, milk, vegetables and fruits. Milk, although examined for the proper quantity of solid fats, etc., is not subject to the microscope in a bacteriological sense. Every large city ought to have competent microscopists to make examinations of raw meat, a great deal of which contains germs perhaps more deadly than the cholera spirillum.

As a warning to young men the statement may be made that some good observers place excessive ambition as an etiological factor of phthisis. Medical men may take this to themselves with profit.

The old controversy about the steaming of milk is again heard, but the city authorities are still very far from enforcing that method of sterilizing.

SKIN GRAFTING.

Dr. Willy Meyer has operated in some cases with remarkable results. One is brought to mind where the nose and eye had been destroyed by fire. The interesting point is the painting of an artificial eye on the headed graft. The flap was taken from the temporal region and grafted to form the whole of the side of the nose, and to cover over the right orbital cavity. The operation was entirely successful, and may occupy a place in the annals of "artistic" surgery with ease. A remarkable operation in grafting was performed in the city about thirteen years ago. The nose had previously been destroyed by fire, the bones and cartilages being absent; great ingenuity was shown in the formation of a new nose from the middle finger of the left hand. The steps were as follows: removing the nail, the matrix was destroyed by acids and the finger denuded of skin at its distal extremity; it was then inserted into a pocket formed in the skin and tissues covering the frontal bone near the interial angular processes; the sides of the nose were formed by a series of small flaps which dovetailed the one into the other. At the end of ten weeks the finger was amputated at its proximal extremity to be left at the site of the nose; the resulting organ was of a type distinctly Roman, the knuckle forming the bridge, and it altogether presented a very nice appearance. Perhaps it would not have been lauded as a beauty, but compared in a general way with any number of noses, it was not bad looking. The view has been advanced that in all operations of grafting to form a nose, it would be a very safe procedure to support the organ by a flap from the upper lip. This may be a good method in women, but a man would hardly care to have a hirsute appendage from his nose.

*Papier-maché* still holds first place as a material for artificial noses.

### RESECTION OF THE HIP JOINT.

A very interesting discussion was recently held at the Academy of Medicine on this subject, in which the question of artificial joints occupied a prominent position. Dislocations of long standing were particularly referred to. The presentation of cases showed two very interesting ones in both of which very excellent results were obtained from the operation. The greater number of surgeons present believed that of the two methods, *i. e.*, 1st, removal of the head, neck and tuberosity of the femur; 2d, removal of the head alone, the latter was to be preferred. Reference was made to means of reduction prior to operation, tenotomy being advocated, but the experienced opinion was that it is rarely of benefit as only the superficial muscles can be divided.

An increase in thickening of the periosteum at the sight of the false joint was considered an advantage rather than otherwise.

### EDUCATION.

A great deal of dissatisfaction has been manifested recently in New York, owing to the fact that in some of our schools young, though brilliant men, have been appointed to professorial chairs, who, although they teach well, rely on the experience and work of others. Teachers of large numbers of men should be [illegible]le to give either in the ecture room or in the amphitheatre, their own personal experience, not the words of others, which the student can find in his library. No sensible man will cavil at lack of age alone.

In science age is not a landmark as applied to the scientist, but work and experience is, and an instructor cannot teach what he does not know, and it is only experience which will tell him. This is applicable to other cities than New York, with perhaps more concentration. Very good advice to give a student is, go to the fountain head for knowledge if possible.

Writing of college work brings to mind that the voice of rumor says, that two of the representatives of the city have decided to increase the time requirement to four years, dating from matriculation or regent's examination; if this is a fact, it will undoubtedly increase both the quantity and quality of scientific work done by graduates.

The expressed sentiment of the foremost medical men of the State show that they nearly all favor increase of time requirement. The English system would perhaps be an improvement to the present one, that is, counsels or boards controlled by one constitution.

It may be of interest to those not familiar with the *modus operandi* of examining candidates in England to know that extraordinary precautions are taken against cribbing or any form of cheating, which alas is liable to occur at almost any examination.

Candidates having registered sometime before and being duly accepted repair on the appointed day to the examination hall, where the examiner awaits him and his fellows. The door being locked the identity of each man is lost; he merely represents a number. The papers containing the questions, written by the counsels one, two or three hundred miles away, are opened by the examiner and distributed. The candidates having finished their papers, the examiner seals them, and they are passed out through an orifice, to be instantly mailed before the door is opened.

The work is judged by men who perhaps have never seen the candidates. With such strict precautions it is almost impossible for the candidate to take advantage of the examiner in any way.

### CLUB-HAND.

Dr. Sayre, Jr., presented a case at the Academy where several bones of the carpus were absent, the hand flexed at the wrist and the forearm fixed in a position of supination. The interesting point, was the proposed splitting up of the lower end of the radius, and attaching it in some way to the carpus, giving in this way a fixed but useful joint.

### GONORRHŒA.

The old method of treating gonorrhœa by insufflation has been revived at the DeMilt Dispensary by Dr. Chetwood. The urethral canal being expanded by a speculum, Brown's being the best for this purpose, the insufflator used is supplied with a bulb, on a pressure of which the drug is thrown into the canal in the form of a fine light cloud.

Various preparations have been used, but stearate of copper seems to be attended with very excellent results, particularly in inflammation of a more or less chronic character, where it seems to stimulate the mucous membrane to the secretion of a healthy mucous. It has the advantage over the injections that the physician can always see the operations carried out himself, and the entire surface on the mucous membrane being put on the stretch by the speculum is exposed to the influence of the drug.

R. HARCOURT ANDERSON.

260 *West 21st Street, New York City.*

